# Mobile Decision Support Tool for Emergency Departments and Mass Casualty Incidents (EDIT): Initial Study

**DOI:** 10.2196/10727

**Published:** 2018-06-22

**Authors:** Nicholas Boltin, Diego Valdes, Joan M Culley, Homayoun Valafar

**Affiliations:** ^1^ Department of Computer Science and Engineering University of South Carolina Columbia, SC United States; ^2^ College of Nursing University of South Carolina Columbia, SC United States

**Keywords:** triage, mass casualty incidents, decision support tools, mobile technology, biomedical informatics, human-computer interaction

## Abstract

**Background:**

Chemical exposures pose a significant threat to life. A rapid assessment by first responders and emergency nurses is required to reduce death and disability. Currently, no informatics tools exist to process victims of chemical exposures efficiently. The surge of patients into a hospital emergency department during a mass casualty incident creates additional stress on an already overburdened system, potentially placing patients at risk and challenging staff to process patients for appropriate care and treatment efficacy. Traditional emergency department triage models are oversimplified during highly stressed mass casualty incident scenarios in which there is little margin for error. Emerging mobile technology could alleviate the burden placed on nurses by allowing the freedom to move about the emergency department and stay connected to a decision support system.

**Objective:**

This study aims to present and evaluate a new mobile tool for assisting emergency department personnel in patient management and triage during a chemical mass casualty incident.

**Methods:**

Over 500 volunteer nurses, students, and first responders were recruited for a study involving a simulated chemical mass casualty incident. During the exercise, a mobile application was used to collect patient data through a kiosk system. Nurses also received tablets where they could review patient information and choose recommendations from a decision support system. Data collected was analyzed on the efficiency of the app to obtain patient data and on nurse agreement with the decision support system.

**Results:**

Of the 296 participants, 96.3% (288/296) of the patients completed the kiosk system with an average time of 3 minutes, 22 seconds. Average time to complete the entire triage process was 5 minutes, 34 seconds. Analysis of the data also showed strong agreement among nurses regarding the app’s decision support system. Overall, nurses agreed with the system 91.6% (262/286) of the time when it came to choose an exposure level and 84.3% (241/286) of the time when selecting an action.

**Conclusions:**

The app reliably demonstrated the ability to collect patient data through a self-service kiosk system thus reducing the burden on hospital resources. Also, the mobile technology allowed nurses the freedom to triage patients on the go while staying connected to a decision support system in which they felt would give reliable recommendations.

## Introduction

Biomedical informatics is an interdisciplinary field that deals with the storage, retrieval, sharing, and optimal use of data and knowledge for problem-solving and decision-making. Historically, Health Information Systems (HIS) and the medical community, in general, have been slow to adapt to new technologies [1]. However, healthcare institutions are now seeking to develop integrated computer-based information management environments with various informatics tools that aid care-givers in decision-making [2].

One area of healthcare that could benefit from an integrated decision support system is the hospital emergency department (ED). The ED typically operates under a set of conflicting main objectives. On the one hand, the ED system aims to process patients promptly, and on the other hand, the most optimal treatment of patients relies on a collection of detailed information from patients, which is time-consuming. The net effect of these competing objectives results in a compromise in one of the two main objectives. Under extreme circumstances like mass casualty incidents (MCIs) where the ED is inundated with many patients, additional constraints are imposed by overwhelmed hospital resources. Adaptation of modern technology can assist in diminishing the degree of compromise during the normal ED operations, and ED operations under MCI conditions.

Over the past few years, a limited set of software products have been presented spanning mobile devices, desktop computers, and Web-based services. Relevant to this study, the National Library of Medicine has created the Wireless Information System for Emergency Responders (WISER) [3], which allows emergency personnel to identify a list of possible chemical substances based on observed patient signs and symptoms. The US Department of Health and Human Services has developed another software tool, the Chemical Hazards Emergency Medical Management-Intelligent Syndromes Tool (CHEMM-IST) [4], which aims to identify a possible syndrome based on observed patient symptoms. Although such software makes significant strides in assisting the process of emergency care, they are not designed for a hospital ED. Therefore, the software efficiency, especially during MCI events, has not been well established [5].

In this report, we present the Emergency Department Informatics Computational Tool (EDICT), a comprehensive tool for processing, management, and triage of patients during an MCI. EDICT is designed to assist with the process of seamless data collection, aggregation, and dissemination using mobile technology to facilitate a client-server transaction model. EDICT has also been designed to include a recommendation decision support system, which we have utilized its potential for chlorine exposure. In this report, we present the EDICT software package and demonstrate its efficiency and agreement among nurses in application to a simulated reenactment of a 2005 chlorine spill that took place in Graniteville SC.

## Methods

### Background on Triage Systems

Triage is used to define how patients are categorized in the ED based on the severity of their condition. A triage nurse typically assigns a triage level with little information and in a short amount of time. Therefore, an effective triage requires a complex clinical decision based on small amount of data with a very limited margin for error. Given the complexity of the pragmatic cost of mistakes in patient assessment, triage-nurses typically favor over-triaging patients to guarantee patient care. Triage bias may be tolerable during normal ED operations, but over-triaging patients during an MCI event can place an unnecessary burden on already taxed hospital resources and reduce patient outcome [6,7].

Over the years, many models have been developed for triaging patients at the scene of the incident (field-triage) and in the hospital system (hospital-triage). Most of these models either use a three-tiered color system such as Sort, Assess, Lifesaving Interventions, Treatment or Transport (SALT) [8] or a five-tiered numeric system such as the Emergency Severity Index (ESI) [9]. The ESI algorithm is one of the most commonly used triage systems and is found in over 70% of large hospitals across the United States [10]. Triage algorithms are simplistic to train ED personnel quickly and simplify the decision-making process. However, the simplistic nature of these triage systems is not a reflection of their ability to optimize patient outcome. In fact, the effectiveness of these triage models to accurately triage patients in an MCI is widely unproven [11-13].

A modern triage system should incorporate existing mobile technology to reduce the cost of data collection and improve efficiency by providing rapid and accurate decision support. In the following sections, we outline a prototype for a patient management triage system that can provide decision support for ED personnel during a chemical MCI. This innovative tool utilizes mobile technology, giving staff the freedom to move about the ED, provides secure data collection with redundant features, and deploys artificial intelligent (AI) algorithms to provide clinical decision support.

### EDICT: Emergency Department Informatics Computational Tool

EDICT was designed to improve patient outcomes during a chemical MCI through the utilization of mobile technology and incorporation of AI. To achieve its objectives, the EDICT software package integrates three main components: (1) fast and accurate data collection through aggregation and dissemination of information; (2) re-engineering of the patient processing protocol; and (3) a clinical recommendation system. Each of these components is described in the following sections.

#### Component 1: EDICT Data Collection, Aggregation, and Dissemination Platform

The EDICT software package was engineered to seamlessly facilitate data collection, aggregation, and dissemination during an MCI event. EDICT employs a Client-Server model that allows safe and fast bidirectional communication of data between mobile devices and a data storage server. The data-storage and AI servers can be located offsite to ensure additional data security. In addition to the centralized server, each client device creates and maintains its local database. This concurrent model of distributed and centralized data storage provides data redundancy that ensures data integrity against hardware failure. Recovery from a server-crash can be accomplished through aggregation of all the local data distributed across the client’s local database. In return, local data can be reconstituted from the central server in the case of accidental damage to a client device.

Another critical feature of EDICT is providing situational awareness to all the pertinent members of the ED personnel. The current implementation distributes relevant information to all mobile devices such as the number of patients admitted, number of critical and noncritical patients, and geographical distribution of admitted patients. It is easy to envision future expansions of this feature to include a list of available ED resources and occupied resources as part of the global situational awareness report.

**Figure 1:**
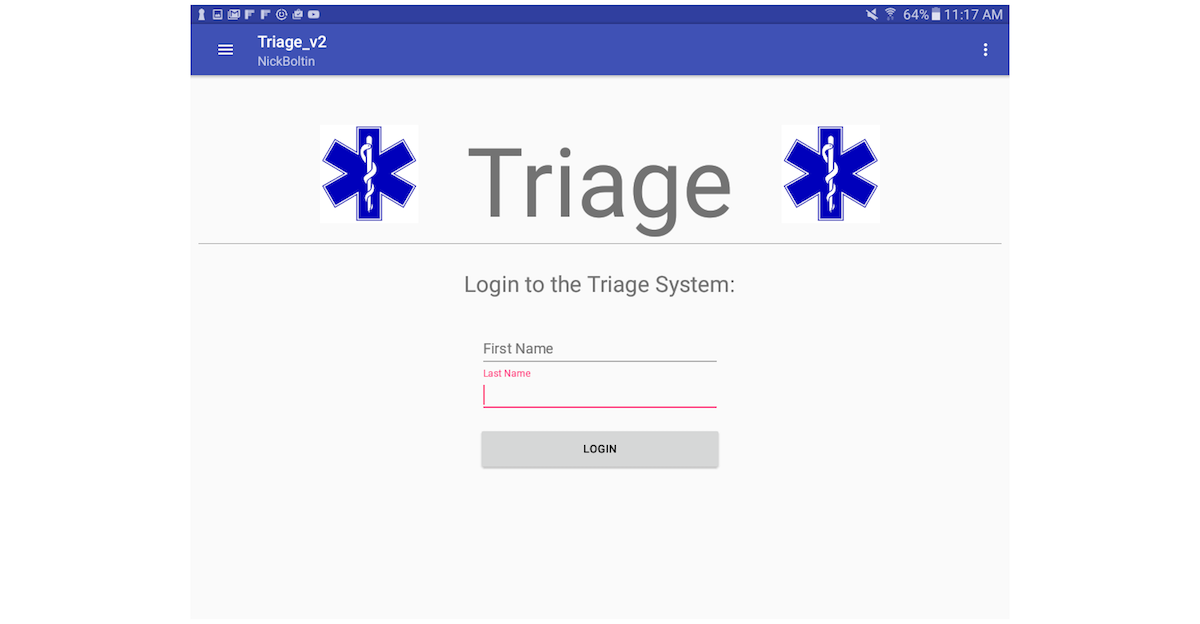
Triage app home screen.

**Figure 2:**
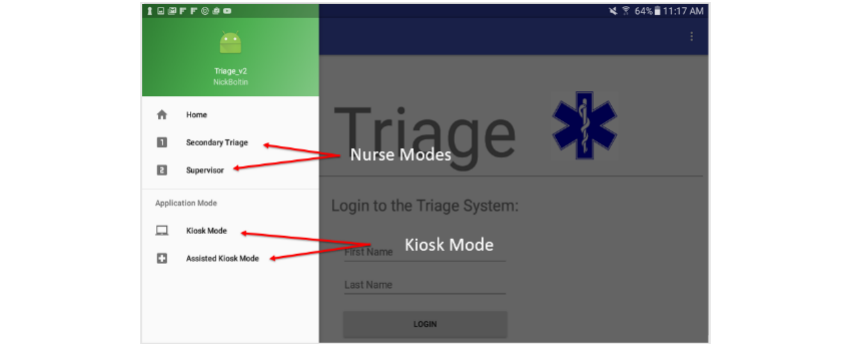
App navigation and set-up.

The current version of the application allows the proper function of each device to be selected through a login and setup process (Figure 1). A super-user can select between two distinct modes of operation: patient mode and nurse mode (Figure 2). The ability to switch between the two modes provides a dynamically adaptive system that can mitigate the effects of a surge at any point of the patient processing pipeline. Each of the two modes of operation will be described in sections below.

##### EDICT’s Mobile App: Kiosk Mode

The kiosk mode enables a kiosk system that facilitates the process of collecting data from patients and divides into two operational submodes: assisted and nonassisted. The nonassisted mode will initiate the kiosk data collection module and can be operated by a patient. The assisted mode is identical, with the exception that the login identification of the assistant ED personnel is recorded.

**Figure 3:**
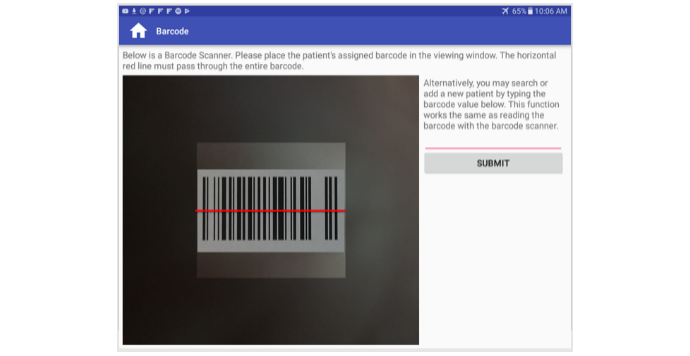
Kiosk barcode scanner.

When patients interact with the kiosk system, they are greeted with a welcome screen and asked to scan their barcode (Figure 3). Instructions are given on how to correctly align the barcode inside the scanner window. Under some abnormal conditions, the barcode scanning may fail or take too long. To mitigate such instances, patients and nurses have the option of entering the numeric value of their barcode to bypass the scanning process.

**Figure 4:**
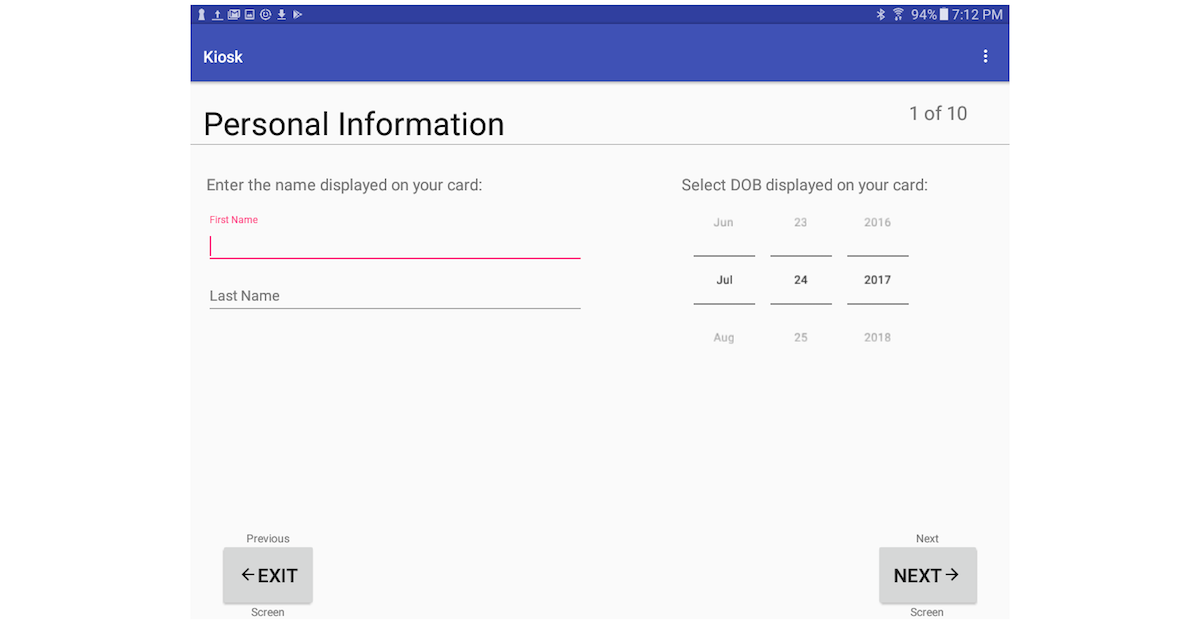
Kiosk demographic screen.

**Figure 5:**
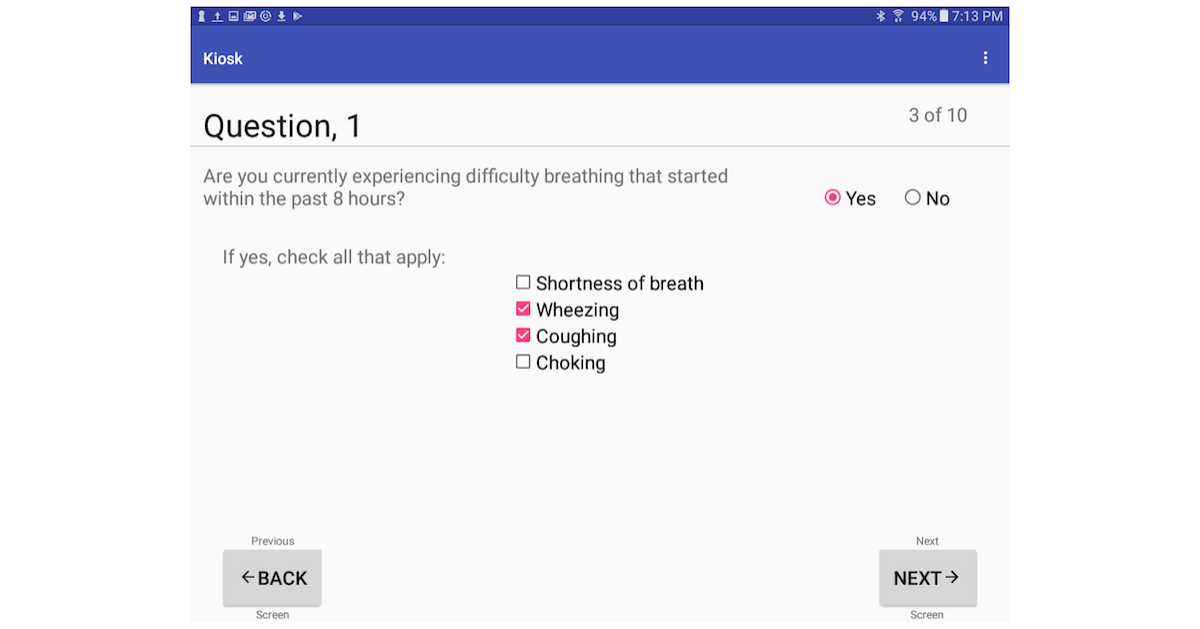
Kiosk sign/symptom screen.

**Figure 6:**
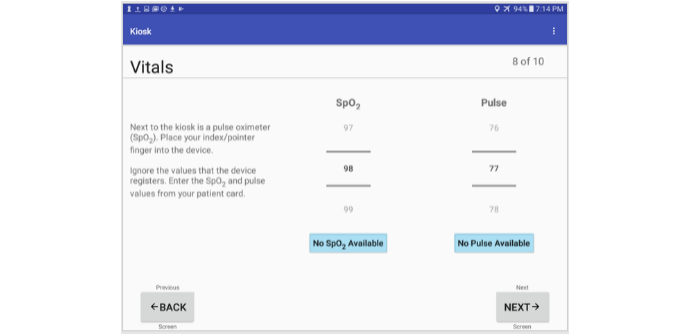
Kiosk patient vitals screen.

**Figure 7:**
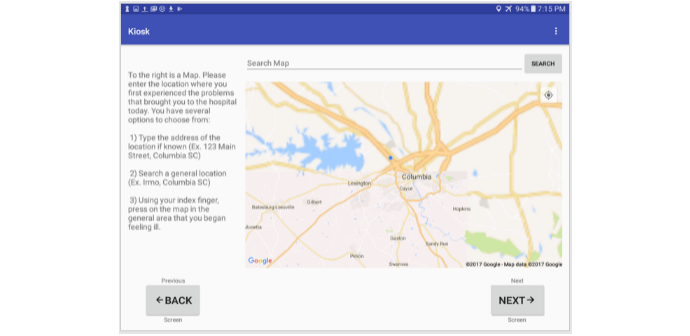
Kiosk google map screen.

On the patient’s initial entry into the system, the central server creates an instance of a new record based on barcode values. Patients then proceed linearly through a series of screens that collect information on their demographics including name, and date of birth (Figure 4). Information related to their symptoms and chief complaint (Figure 5) are also collected. Additional features of the kiosk system include collecting pulse rate and oxygen saturation values using a pulse oximeter (Figure 6). The geographic location where a patient first experienced their signs or symptoms (Figure 7) is also collected. Google maps Application Programming Interface [14] facilitates the location and can accept a street address, a manually placed marker, or longitude and latitude.

##### EDICT’s mobile application: Nurse Mode

**Figure 8:**
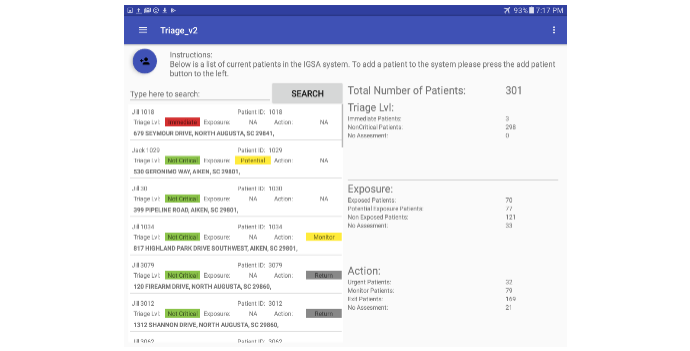
Nurse global view screen.

**Figure 9:**
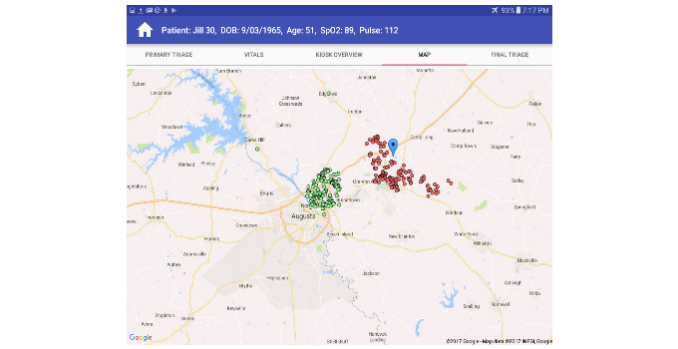
Nurse google map screen.

The nurse mode provides more diverse subfunctions when compared to kiosk mode. One example is the information related to global awareness of the MCI event. The situational awareness view (right panel, Figure 8) gives an overview of the event by displaying the number of patients in the system and a breakdown of triage levels currently assigned. The spatial view (Figure 9) helps establish the geographical scattering of patients within the event which is critical when determining if incoming patients have been exposed to the MCI event.

**Figure 10:**
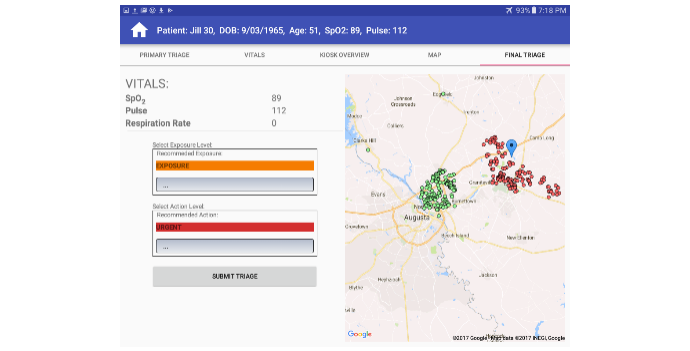
Detailed patient information screen.

The nurse mode can also be used to view a comprehensive list of patients currently in the system and a summary of collected information (left panel, Figure 8). Detailed information can be displayed by selecting an individual patient in one of three ways: (1) manually navigating the list of patients, (2) using the search dialogue, or (3) scanning a patient’s barcode. Figure 10 illustrates an example of the detailed patient information screen. Additional functions are available through different functional tabs at the top of the screen and include: review or update patient data such as geographical location, signs and symptoms or initial triage category. Tabs are also available for reviewing AI recommendations for each patient (subject to availability of sufficient data), and the evaluation screen, where nurses assign the final triage classification. EDICT’s system menu (top left corner Figure 8) allows easy navigation to other modes or screens.

#### Component 2: Re-engineered Patient Processing Pipeline

**Figure 11:**
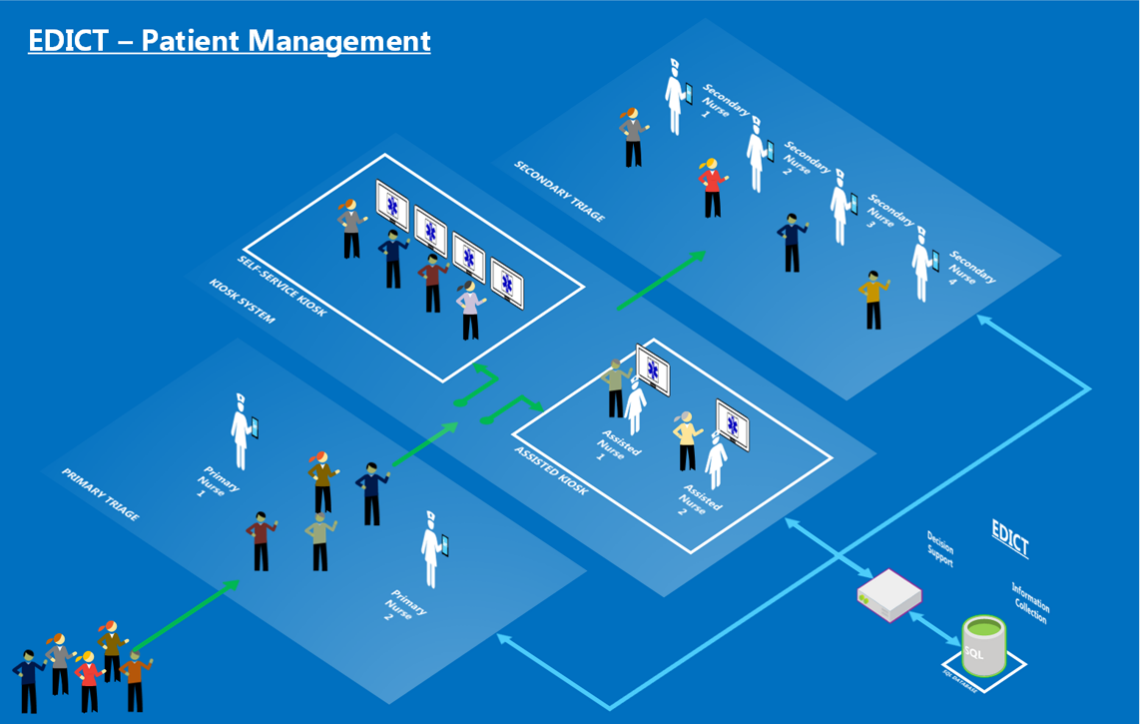
EDICT, a mass casualty incident–specific triage tool used to map data gathered by the mobile application to the Irritant Gas Syndrome Agent algorithm. Information gathered in the primary triage, the kiosk system and secondary triage is used to determine a patient's specific exposure level and action.

An improved patient management system can benefit from establishing order during the chaos that takes place during an MCI. Here we propose a patient processing pipeline that helps improve patient management while facilitating a faster mechanism for collecting data and tracking patients. The patient tracking system will consist of three main stages shown in Figure 11. The three stages are denoted as the “primary triage,” “kiosk system,” and the “secondary triage” phases, which are described in the following sections.

##### Primary Triage

The main objective of primary triage is to identify the patients who need immediate care. Functionally, ED personnel can engage the arriving patients in a variety of ways. For our research, we assume patients will be given a wristband with a barcode that will serve as the patient’s unique identification for the remainder of their virtual existence within the EDICT system. In addition to receiving a wristband, patients will be evaluated by a primary triage nurse if necessary and receive a triage category of “Immediate” if assessed to have a life-threatening problem and sent directly for treatment. All remaining patients are initially categorized by default as “not critical” and directed to the kiosk area for further acquisition of information.

##### Kiosk System

The kiosk system is designed to interactively collect individual information such as name, date of birth, and other demographics from patients initially categorized as “not critical.” Additional information is obtained to help define the location of the incident using an applet like Google maps. Data is also collected on signs and symptoms of the presenting condition, and chief complaint. The kiosk stage is partitioned into assisted and nonassisted sections, where patients can complete the registration process independently or with the help of designated ED personnel. The patient information is gathered concurrently by multiple mobile devices and can, therefore, contribute to rapid data collection and patient processing.

Information collected from patients is aggregated into a central database and analyzed by the AI system to understand the nature of the incident better and provide decision support for triage recommendations. The aggregated information is also disseminated throughout the system to all registered ED personnel as a means of providing a global view of the event. After patients have completed the data collection process, they are given instructions to proceed to the final stage of the patient management system, secondary triage.

##### Secondary Triage

At Secondary Triage, nurses are tasked with providing the most appropriate triage category to optimize patient outcomes. EDICT assists secondary triage nurses by providing decision support specific for each patient. EDICT offers a complete information profile and a system triage recommendation based on the AI analysis of each patient. The secondary-nurse can scan the patient’s barcode to retrieve information collected, which eliminates errors related in the miss-identification of patients. The nurse can view recommendations from the central server on a patient’s possible chemical exposure, and the appropriate course of action for each patient. The nurse provides the final triage category by agreeing or disagreeing with the decision support system recommendation and providing a rationale when they disagree. The AI recommendation system is described in the following section.

**Table 1:** Summary of the decision logic for the triage recommendation system. Nurses are given recommendations by the decision support system based on information provided by patients in the kiosk system.

Category			Outcome
**Exposure**			
	Exposed	Patient has been exposed to an IGSA	
	Potentially exposed	Patient has potentially been exposed to an IGSA	
	Not exposed	Patient has not been exposed to an IGSA	
**Action**			
	Exit	Retriage using a nonchemical related algorithm	
	Monitor	Monitor the patient for up to 8 hours for latent symptoms	
	Urgent	Seek immediate medical treatment	

#### Component 3: Triage Decision Support System for Irritant Gas Syndrome Agent Exposure

EDICT is designed to provide clinical decision support for each patient based on available information. EDICT offers a summary of all data acquired for each patient as they proceed through the patient processing pipeline. When sufficient information is gathered for a given patient, the central AI engine in EDICT provides inferred recommendations regarding a patient’s exposure level, and the most effective course of action for each patient. The patient exposure feature is designed to separate patients who visit the ED uninvolved in the MCI event and therefore do not need to be subjected to the chemical triage process.

The current recommendation system of EDICT is optimized for exposure to an Irritant Gas Syndrome Agent (IGSA; Figure 1) [15]. However, in principle, EDICT could house a comprehensive collection of possible triage mechanisms from which the optimal procedure could be selected for each MCI. Table 1 describes the categories for exposure and the recommended actions that are provided by the central AI engine in EDICT based on the IGSA mechanism.

### Test and Evaluation Process

In April of 2017, a large-scale exercise was conducted utilizing over 500 emergency responders and nursing students. For this exercise, a chemical MCI event was simulated to replicate a derailed train accident that took place in 2005, releasing chlorine gas into the town of Graniteville, South Carolina. Participants were separated into 4 groups: patients, assisted kiosk helpers, primary triage nurses, and secondary triage nurses. EDICT was used for patient management, data collection, and decision support.

During the exercise, 15 tablets were used to study the effectiveness of the patient management system. The tablets were partitioned into 3 functional groups based on the app’s operational mode: assisted-kiosk mode, non-assisted-kiosk mode, and nurse mode. EDICT was evaluated on its efficiency in triaging patients and the agreement with the decision support system. Information related to each of the participant groups and EDICT users is found in the following sections

#### Emergency Department Patients

**Figure 12:**
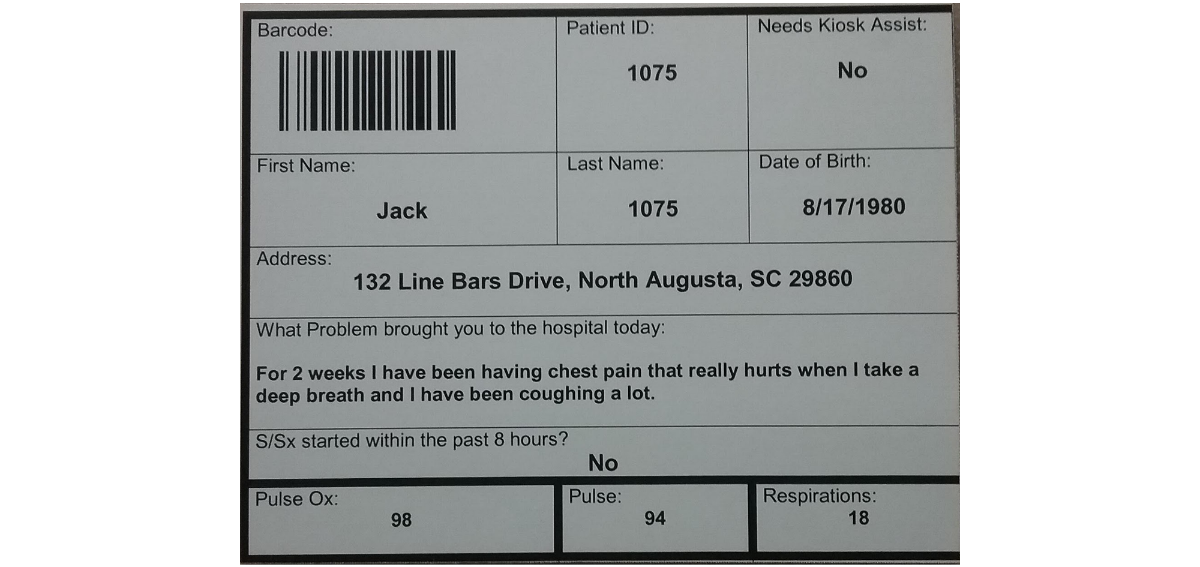
Example of a patient card given to participates in the chemical mass casualty incident exercise. Participates were asked to enter information and answer questions in the kiosk system based on the cards they received.

Two hundred ninety-six students from USC’s nursing program participated as ED patients. Of the participants, 95% were female and 90% were 18-24 years old. ED patients were split randomly into 2 patient populations. The first group consisted of 198 patients that were part of the chlorine exposure event. Data used for this group was gleaned from de-identified medical records of patients from the 2005 train derailment. The second group consisted of 100 patients suffering from ailments unrelated to the MCI event. The data for this group was acquired from de-identified medical records of patients with flu-like symptoms who visited the same hospital in 2016. As part of the exercise, students randomly received a patient card (Figure 12), for either a victim of the first group or a flu patient from the second group. The cards outlined specific information related to their visit to the ED, vitals, and a location where they first felt sick. Students used the information displayed on their card to interact with the kiosk system and proceed through the patient processing pipeline. Students had no pre-exercise access to their patient data or the EDICT software until they entered the simulated ED.

#### Kiosk Helpers

Five assisted kiosk stations were set up for the April 2017 exercise. Each station was assigned a kiosk helper tasked with assisting patients with entering information into the kiosk system. The helpers were all female between the ages of 29-59. They received 1 hour of individual training before the exercise with a member of the app development team who guided them with navigating through the kiosk system and entering patient information.

#### Triage Nurses

Thirteen registered nurses and emergency responders were assigned to evaluate patients in the secondary triage stage. There was 1 male nurse, and 12 female nurses between the ages of 30-69. Each received 1 hour of training before the exercise with a member of the app development team on how to use the nurse-interface. They also received instruction on how to review patient information using the app and how to assign triage categories based on the IGSA algorithm. In addition, secondary-nurses were given an information packet describing the IGSA algorithm and the MCI scenario.

### Data Exclusion

Two categories of data were excluded from our analysis of EDICT’s performance. The first consisted of records that contained No Available (NA) information. Some NAs were identified as “Immediate” patients who required instant attention and were removed from the patient pipeline or patients who were able to bypass a section of the registration process. The latter cause is currently under investigation by the development team and will be resolved in a future iteration of the app. In total, an insignificant number of NA instances were observed (214/5096, 4% of database transactions) and therefore have little impact on our outcomes.

The second criterion for data exclusion was based on the implausibility of data values (outliers). Outliers were identified using the Tukey’s method described in Equation 1 below, where q is a tabulated score [16], w is the range of the normal distribution and s is the standard error of the sum of the means. The Tukey’s test uses the interquartile range (IQR) defined in Equation 2 below to identify outliers and removing points +/-1.5*IQR. Outliers were identified for each of the questions in the kiosk, the time spent at the kiosk, the time patients spent waiting to enter secondary triage and the time spent in secondary triage. The exclusion of this category of data is justified by students who may have received a phone call or engaged in a chat discussion on their cell phone during the exercise. Other more relevant exclusions are based on patients who may have needed to pause the registration process for personal reasons (eg, bathroom break).

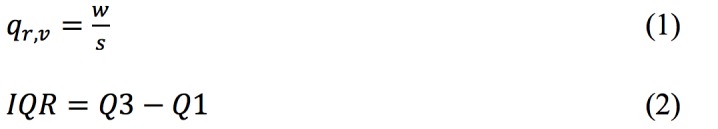



## Results

### Component 1: EDICT Data Collection, Aggregation, and Dissemination Platform

During the April 2017 exercise, every item of submitted data and its corresponding timestamp was captured in EDICT’s central database. The information included: patient demographics, answers to all the Kiosk questions, vitals, illness onset location, the central server’s recommended triage, and triage levels assigned by nurses, to name a few. In summary, the EDICT software package captured 5471 data transactions for the April 2017 exercise.

**Figure 13:**
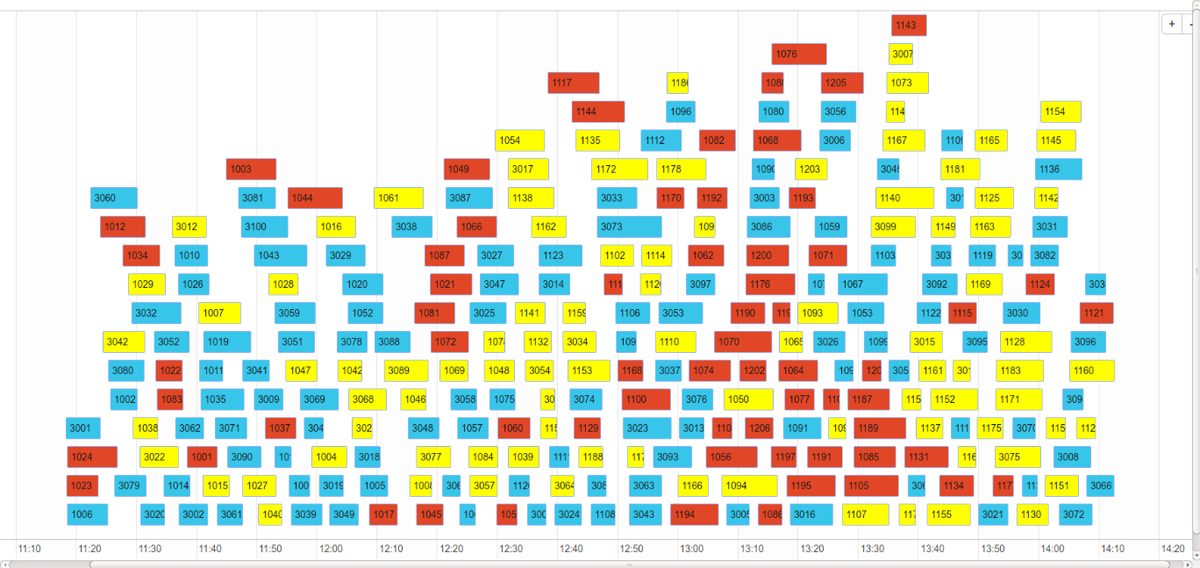
Overall triage results from the April 2017 exercise. Blue cells indicate patients EDICT recommended as not exposed. Yellow cells indicate patients EDICT recommended as potentially exposed and red cells indicate patients EDICT recommended as exposed. The length of the cells describes the amount of time the patient spent in the patient management system.

The patient management utility of EDICT processed 296 patients within a window of less than 3 hours. This results in an average of 36 seconds per patient to complete the initial triage, information collection, waiting to be seen by a secondary-triage nurse, and the final triage assessment. The information acquired by the data aggregation mechanism of EDICT can provide a global view of the event as illustrated in Figure 13. In this figure, each block represents the interval of time required to process each patient. The blue, yellow and red cells in Figure 13 correspond to patients categorized by EDICT as not exposed, potentially exposed, and exposed respectively.

### Component 2: Re-engineered Pipeline of Patient Processing

The second component of EDICT aims to improve individual patient’s processing time and patient management. Timestamps captured by EDICT have been used to assess the efficiency of each step and identify outliers. By analyzing the outliers found at each of the data points we could identify areas of concern and investigate technical or usability issues. The following sections provide results related to each of the three components of the patient processing pipeline.

#### Kiosk System

**Figure 14:**
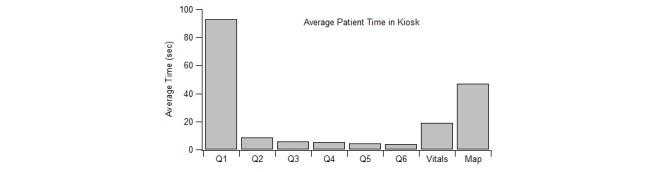
Time comparison of questions asked in the patient kiosk system.

The efficiency of the kiosk system and its discrete question components were measured using timestamps from patients as they progressed through the questionnaire screens. Table 2 summarizes the results of our analysis with and without outliers. In this table, the first column corresponds to the different questions asked in the kiosk system. The second column indicates the number of excluded patients from the 296 created patient IDs. Figure 14 shows the average time spent by patients answering each question in the kiosk system. Of the 296 created patient IDs, 288 completed the kiosk after removing outliers. On average, patients required 3 minutes, 22 seconds to complete the patient kiosk system. The longest and shortest completion times consisted of seven minutes, 12 seconds and one minute, eight seconds respectively. Question 1 required the longest completion time with an average of 92.9 seconds closely followed by the Google map with an average of 46.9 seconds. Questions with only checkboxes (Questions 2-6) required the least amount of time to complete with question 6 being the shortest average of 3.7 seconds.

#### Secondary Triage

Efficiency in the secondary triage stage was measured by examining two factors: the wait time separating the kiosk and the secondary triage stages, and the duration of the secondary triage stage. Table 3 summarizes the average, maximum, and minimum time required by patients to complete various portions of the triage process. The triage completion time in Table 3 corresponds to the time it took patients to complete the entire process, starting from the first entry into the system until the exit from the secondary-triage stage.

### Component 3: Triage decision support system for Irritant Gas Syndrome Agent Exposure

While patient processing speed is an essential aspect of a patient management system, it should be at no cost to improving patient outcome. Therefore, it is as equally important to review the performance of the AI recommendation system. The app’s decision support system was quantified by examining the agreement and disagreement between secondary nurses and the decision support system regarding patient exposure and triage action (Tables 4 and 5). The data shows that 286 of the starting 296 patients (96.6%) completed the triage process and received recommendations from EDICT. In summary, EDICT’s exposure and action recommendation exhibited 91.6% (262/286) and 84.3% (241/286) agreement with nurses’ assessments, respectively. It is worth noting that in the critical subcategory of patients requiring Urgent care, there was 100% (11/11) agreement between EDICT’s recommendation and nurses’ assessment.

**Table 2:** Summary of data outliers' (N=296) time spent with each question. Strict rules were developed by identifying outliers at each step of the triage process. These outliers were then investigated further to see if a user or technical error could be determined.

Step	Outliers, n (%)	Mean of Outliers (sec)	Mean with Outliers (sec)	Mean without Outliers (sec)
Q1	2 (1)	240.50	94.04	92.95
Q2	19 (7)	30.89	10.31	8.76
Q3	20 (8)	26.45	7.05	5.63
Q4	14 (5)	16.57	5.71	5.17
Q5	6 (2)	15.83	4.42	4.18
Q6	11 (4)	13.18	4.03	3.67
Vitals	19 (7)	65.26	21.86	18.82
Map	10 (4)	166.90	51.07	46.93
Waiting	25 (12)	1095.84	156.84	45.58
Time in kiosk	1 (1)	593.00	203.76	202.41
Time in secondary	14 (7)	202.50	77.79	69.56

**Table 3:** The mean, maximum, and minimum amount of time a patient spent waiting to be seen by a nurse, in secondary triage and the overall time to be triaged using EDICT.

Stage	Mean (sec)	Min (sec)	Max (sec)
Wait time	45	0	117
Secondary triage time	69	19	168
Triage complete time	334	152	646

**Table 4:** Exposure agreement among secondary triage nurses and the decision support system for the Irritant Gas Syndrome Agent triage.

Nurse Input	Computer Recommendation		
	Exposed (n)	Potential (n)	Not Exposed (n)
Exposed (n)	65	8	1
Potential (n)	1	80	0
Not Exposed (n)	2	12	117

**Table 5:** Action agreement among secondary triage nurses and the decision support system for the Irritant Gas Syndrome Agent triage.

Nurse Input	Computer Recommendation		
	Urgent	Monitor	Exit
Urgent	11	10	11
Monitor	0	57	23
Exit	0	1	173

## Discussion

During the April 2017 exercise, kiosk helpers and triage nurses each received 1-hour of training. This was necessary to achieve familiarity with the system. The current version of EDICT was designed to focus on efficiently triaging patients related to an IGSA. Future development will look to create decision support for additional MCI scenarios and deploy EDICT during normal hospital operations. Everyday use of EDIT by caregivers would minimize the amount of training necessary for its deployment during an MCI.

### Principal Findings

The utility of EDICT evaluated during a large-scale mock exercise demonstrated many successful aspects of the system. The efficiency of such an approach has the potential to substantially improve patient management during chaotic situations, improve patient outcome, and provided a research platform for data collection, data-mining, and modeling during an MCI related triage. In Table 2 we have presented information related to outliers in each stage of the patient triage process. While in this work we have used these temporal anomalies to further investigate the functionality of the app, during an actual deployment of this app, this feature can be used to monitor patient progress. For example, a patient who may exhibit a long waiting time or does not have an exit timestamp may be traced and any problems rectified. The fast analysis of complex data by computers allows for incorporation of sophisticated triage processes, which will inevitably lead to improved patient outcomes.

Two components of EDICT have contributed substantially to accelerating patient processing. The first component harnesses the organization and improved efficiency of a pipeline mechanism during an MCI event. The utility of a pipeline to improve productivity has been exploited significantly in designing current computer hardware [17] and predates to as far back as Henry Ford’s Model T production [18]. The second contributing factor takes advantage of the concurrency in gathering data and processing patients which demonstrates dynamically adaptive nature of EDICT. This was accomplished by using several mobile devices— as many as eight at times—to gather patient data in the kiosk system and triage patients in the secondary triage stage. Since a given mobile device can function in either kiosk or nurse mode, the utility of the devices can be altered to accelerate the slowest segment of the patient processing pipeline. For instance, during our exercise, from between 12:30 pm and 2:00 pm (Figure 13), a rush of patients inundated the kiosk stage of the pipeline. In response, two additional tablets were switched into kiosk mode and added to the patient processing pipeline to resolve a potential bottleneck. This feature of the app allows for real-time modification to the system to satisfy the most demanding portion of the triage process.

### Limitations

Future iterations of EDICT will look to resolve important obstacles identified during our analysis. First, despite a 97.1% (5174/5328 transactions) data completeness, some patients were able to bypass sections of the software by using the app in unintended ways (eg, exiting the app and reopening it). Second, during the exercise, we identified some instances where the final submission button was not clicked by the user (nurse or patient). These instances were the primary contributors to anomalous times. To resolve these issues, future developments of the app may include automatic time-out features.

A key aspect of developing a triage system is the identification of bottlenecks or areas in which the patient processing might be slowed down. By quantifying the time patients spent at different sections, we were able to identify and remedy these bottlenecks for future iterations of EDICT. For example, patients spent more time on question 1 in the kiosk system than any other question. The expertise of a human-computer interaction researcher can help design better approaches to the limitations imposed by the cumbersome use of the on-screen keyboard. Advances in Natural Language Processing can also be of immense help in this category.

During the April 2017 exercise, we anticipated two additional limitations: battery life, and internet availability. Although both issues are current limitations for any mobile development, they can be resolved in numerous ways. During the exercise, we provided redundancy in our system by having power-packs ready for use if necessary. A backup laptop server with 10 hours of battery life and a battery operated mobile Wi-Fi system was also prepared to handle any possible power failure. Theoretically, with the use of solar panels, one could deploy our independent integrated system in any remote location.

### Conclusions

Analysis of the data from the 2017 drill allowed us to quantify user behavior and measure the performance of the decision support system. The data shows that the kiosk system design performed well during the exercise regarding patient management related to a chemical MCI. Of 296 patient users, 97.3% (288/296) were able to complete the kiosk system either on their own or with an assistant, which suggests very few usability issues. This is substantial considering that participants using the kiosk without an assistant had no training or prior experience using the app.

The data also showed strong agreement among nurses regarding EDICT’s decision support system. Overall, nurses agreed with EDICT 91.6% (262/286) of the time when it came to choosing an exposure level and 84.3% (241/286) of the time when selecting an action. EDICT reliably demonstrated the ability to collect patient data through a self-service kiosk system, thus reducing the burden on hospital resources. Also, the mobile technology allowed nurses the freedom to triage patients on the go while staying connected to a decision support system which they felt would give reliable recommendations. This work has set a precedent for the way patients will be triaged in the future and is a testimony that mobile technology can be a viable resource, even in an environment as chaotic as a hospital ED during a chemical MCI.

## Appendix

Multimedia Appendix 1 Chemical triage algorithm for detecting an Irritant Gas Syndrome (IGSA). The algorithm requires that decisions be made regarding a patient's exposure level and action to correctly assign a triage category.

## Abbreviations

ED: emergency department
EDICT: Emergency Department Informatics Collection Tool
EHR: Electronic Health Record
ESI: Emergency Severity Index
HIS: Health Information Systems
ID: identifier
JIT: Just-In-Time Training
MCI: mass casualty incident
NIH: National Institutes of Health
NLM: National Library of Medicine
NSF: National Science Foundation
SALT: Sort, Assess, Lifesaving Interventions, Treatment/Transport
START: Simple Triage and Rapid Treatment
