# Supplementary figures and images for: Mobile Decision Support Tool for Emergency Departments and Mass Casualty Incidents (EDIT): Initial Study
Source: JMIR Mhealth Uhealth. 2018 Jun 22;6(6):e10727. doi: 10.2196/10727 (PMC6035350; doi:10.2196/10727)

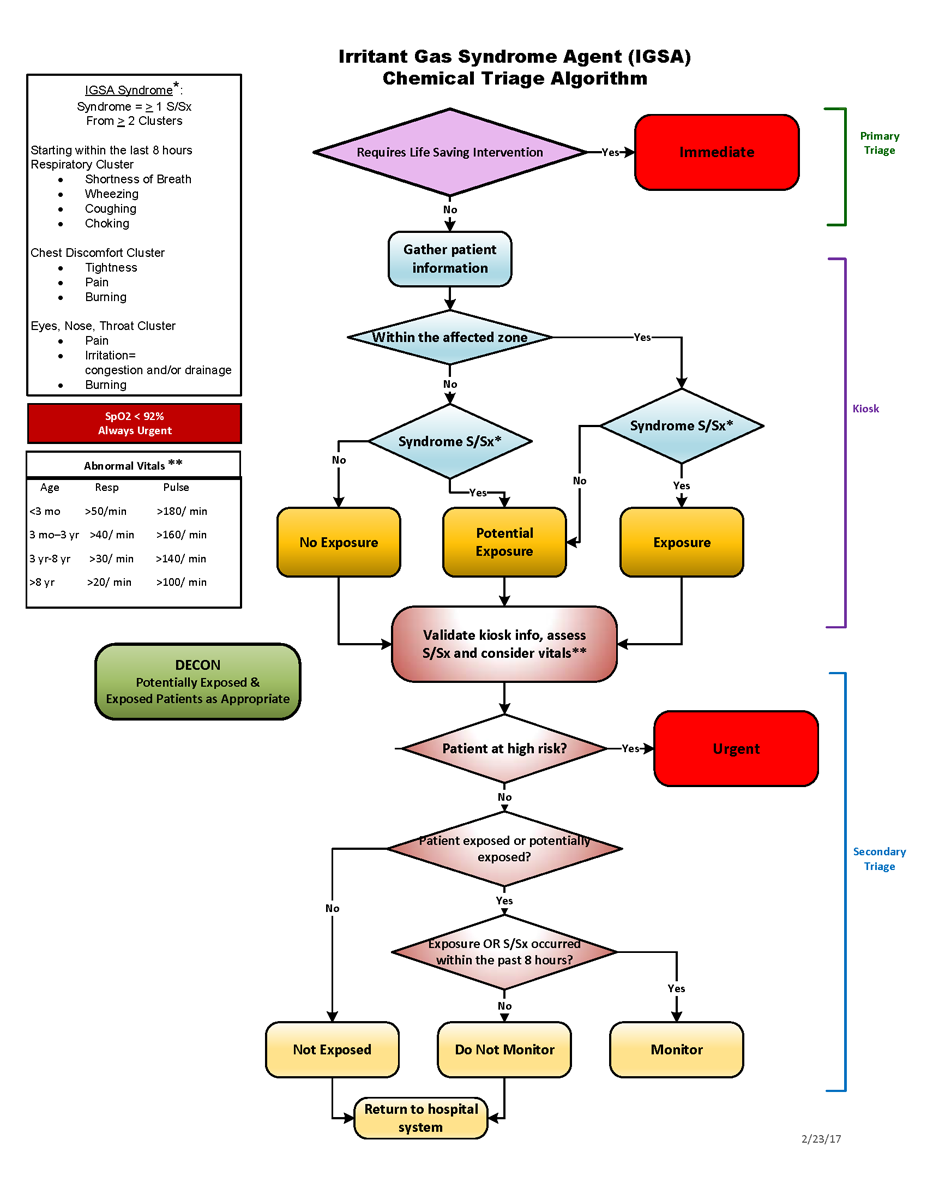

Supplement: Multimedia Appendix 1 [file mhealth_v6i6e10727_app1.png]
